# F-TransR: A sports event revenue prediction model integrating multi-modal and time-series data

**DOI:** 10.1371/journal.pone.0327459

**Published:** 2025-07-16

**Authors:** Guibing You, Kelei Guo, Jie Gao, Hanjie Feng, Wei Zou

**Affiliations:** 1 School of Physical Education and Health, Zhaoqing University, Zhaoqing, China; 2 Financial Department of Zhaoqing University, Zhaoqing, China; 3 School of Computer Science and Software, Zhaoqing University, Zhaoqing, China; University of Queensland - Saint Lucia Campus: The University of Queensland, AUSTRALIA

## Abstract

Sports event revenue prediction is a complex, multimodal task that requires effective integration of diverse data sources. Traditional models struggle to combine real-time data streams with historical time-series data, resulting in limited prediction accuracy. To address this challenge, we propose F-TransR, a Transformer-based multimodal revenue prediction model. F-TransR introduces key innovations, including a real-time data stream processing module, a historical time-series modeling module, a novel multimodal fusion mechanism, and a cross-modal interaction modeling module. These modules enable the model to effectively integrate and capture dynamic interactions between multimodal features and temporal dependencies, which previous models fail to handle efficiently. Experimental results demonstrate that F-TransR significantly outperforms state-of-the-art models, including Informer, Autoformer, FEDformer, MTNet, and CrossFormer, on the Kaggle Sports Analytics and Reddit Comments datasets. On the Kaggle dataset, MSE and MAPE are reduced by 6.4% and 2.9%, respectively, while ${\text{R}}^{2}$ increases to 0.938. On the Reddit dataset, MSE and MAPE decrease by 6.6% and 5.3%, respectively, and ${\text{R}}^{2}$ improves to 0.854. Compared to existing methods, F-TransR not only improves the interaction efficiency of multimodal features but also demonstrates strong robustness and scalability, providing substantial support for multimodal revenue prediction in real-world applications.

## Introduction

With the rapid development of the sports industry, the prediction of sports event revenues has become a key area of research. In the context of abundant data and increasingly powerful computational capabilities, accurately predicting sports event revenues has become an important task for sports organizations, clubs, and related commercial institutions [1]. This task not only helps improve the commercialization efficiency of events, but also provides decision support for event organizers, assisting them in optimizing resource allocation and formulating more effective marketing strategies [2,3]. However, despite the advances in existing studies, traditional methods still struggle with integrating diverse multimodal data, such as real-time data streams and historical time-series data [4]. These traditional models, including regression analysis, ARIMA, and various machine learning techniques, often fail to fully capture the complex, nonlinear relationships between multiple factors like audience numbers, ticket prices, and advertising expenditures, which are crucial for accurate revenue prediction [5]. This limitation becomes even more pronounced when dealing with multi-level and multimodal data, where the performance of these methods often falls short [6].

Despite some existing research attempts to combine multimodal data with time-series data, significant limitations remain [7]. Traditional machine learning methods (e.g., random forests, support vector machines) typically rely on manual feature engineering to handle complex multimodal data, making it difficult to effectively model high-order interactions between different modalities. On the other hand, classical time-series methods, such as LSTM, GAN, and ARIMA, excel at capturing temporal dependencies but struggle to handle multimodal data effectively due to a lack of flexibility and representational power [8,27,28]. Deep learning-based approaches that fuse multimodal data, such as multimodal Transformers or CNN-RNN hybrid models, show strong performance in capturing temporal features within a single modality [9]. However, their fusion of features across modalities often relies on simple techniques like concatenation or weighted averaging, which makes it challenging to capture more complex interactions between data sources [10].

To address these challenges, we propose F-TransR, a Transformer-based model that integrates multimodal data with historical time-series data for improved sports event revenue prediction. F-TransR introduces several novel components, including a real-time data stream processing module, a multimodal fusion mechanism, and a cross-modal interaction modeling module. These innovations allow F-TransR to better handle the dynamic interactions between real-time and historical data, as well as the temporal dependencies that are critical to accurate predictions [11]. The proposed model offers significant improvements over existing methods by addressing the issues related to multimodal data fusion and temporal dependency modeling, providing more robust and scalable solutions for real-world applications [12]. The main contributions of this paper are as follows:

1. An innovative multimodal fusion method is proposed, which leverages the self-attention mechanism of Transformers to effectively integrate multimodal data from sources such as social media sentiment, news reports, and advertising expenditures.
2. A Transformer encoder is employed to model historical time-series data, capturing long-term dependencies and enhancing the ability to process temporal data.
3. A framework combining fusion and prediction modules is designed, which processes the fused features to achieve more accurate sports event revenue predictions. Through these innovations, this research provides a new perspective and method for sports event revenue prediction, with significant theoretical and practical value.

The structure of this paper is organized as follows: Section Related work reviews relevant research, introducing the latest advancements in multimodal data fusion and time-series modeling methods, and compares them with existing approaches, highlighting the innovations of this study. Section Method provides a detailed description of the proposed F-TransR model, including its overall architecture, real-time data stream processing module, historical time-series data modeling module, and multimodal fusion module. Section Experiment presents the experimental design, including datasets, experimental environment and settings, and evaluation metrics, and verifies the effectiveness of the model through comparative and ablation experiments. Finally, Sect Conclusion and discussion summarizes the main contributions of this paper and discusses potential directions for future research.

## Related work

### Sports event revenue prediction research

In recent years, research on sports event revenue prediction has primarily focused on time-series analysis, machine learning, and deep learning methods [13,14]. Traditional statistical models, such as ARIMA (AutoRegressive Integrated Moving Average), perform well with stationary data but struggle with nonlinear relationships and external factors like social media sentiment and advertising expenditures [15]. Machine learning methods like Support Vector Machines (SVM), Random Forests (RF), and Gradient Boosting Decision Trees (GBDT) handle nonlinear data but rely heavily on manual feature engineering and fail with multimodal data.To overcome these limitations, Long Short-Term Memory (LSTM) networks were introduced for capturing long-term dependencies [16]. However, LSTM models primarily work with single-modal data and struggle with managing multiple data sources [17]. Recently, Transformer models, which capture long-term dependencies through self-attention mechanisms, have shown great potential in time-series forecasting [18]. However, they still face challenges in integrating diverse modalities [19].Unlike these models, F-TransR integrates multimodal data, including real-time data streams and historical time-series, improving prediction accuracy and handling the dynamic nature of real-world data [20].

The proposed F-TransR model in this paper addresses these issues by combining multimodal data with historical time-series data and leveraging the Transformer structure to handle the complex dependencies between the two, thus improving the prediction accuracy and generalization ability of the model. F-TransR is capable of processing both real-time data streams, such as social media sentiment and news reports, and capturing long-term dependencies in historical time-series data, enabling precise prediction of sports event revenues.

### Multi-modal data fusion research

With the advancement of deep learning and big data technologies, research on multi-modal data fusion has made significant progress in various fields [9,19]. In tasks like sports event revenue prediction, efficiently integrating data from different modalities has become a key factor in improving prediction accuracy [21]. Recent studies have attempted to incorporate Transformer architectures into multi-modal data fusion, leveraging their powerful self-attention mechanisms to handle long-term dependencies and complex interactions across different modalities. For example, some studies have fused time-series data with BERT or Vision Transformer (ViT), utilizing the Transformer’s strong sequence modeling capabilities and parallel computing advantages to capture relationships between modalities [22]. However, most of these methods focus on single-modal data, such as text or images. While they excel at capturing long-term dependencies, they still struggle with effectively fusing complex multi-modal data. Other studies have explored combining Convolutional Neural Networks (CNNs) and Transformers for multi-modal fusion [23]. While this approach can handle visual features and time-series data well, the fusion mechanisms between different modalities remain insufficient, and computational complexity is high, especially when processing real-time data streams, leading to significant efficiency bottlenecks [24]. Recent innovative work has started to combine Graph Neural Networks (GNNs) with Transformers to model relationships between modalities using graph structures [25]. However, these methods still face limitations in processing real-time data and handling the dynamic fusion of large-scale data streams, often suffering from high computational overheads and difficulty in providing real-time responses [18]. Although existing multi-modal fusion methods perform well in certain scenarios, they are generally focused on static data fusion and lack adaptability and efficiency when it comes to integrating real-time data streams and historical time-series data [26]. The proposed F-TransR model addresses these issues by combining multimodal data with historical time-series data and utilizing the Transformer architecture to efficiently handle real-time data streams while maintaining low computational overheads, providing real-time, accurate predictions of sports event revenues [11].

The F-TransR model proposed in this paper introduces an innovative approach to multi-modal data fusion. It effectively combines the self-attention mechanism and time-series modeling capabilities of the Transformer architecture to address the challenge of effectively merging real-time data streams (such as social media sentiment, news reports, etc.) with historical time-series data. Our model adaptively adjusts the weights of different modalities, capturing complex interactions and long-term dependencies between them, significantly enhancing the accuracy and generalization ability of sports event revenue prediction.

### Time-series modeling research

In recent years, time-series modeling techniques have been widely applied to various forecasting tasks, particularly in fields such as finance, meteorology, transportation, and sports [31,32]. Traditional time-series methods, such as AutoRegressive Integrated Moving Average (ARIMA) and Exponential Smoothing (ETS), perform reliably when dealing with stationary and linear data. However, these methods struggle with complex, nonlinear time-series data, especially in applications where data streams are dynamic and multimodal. To address this limitation, deep learning techniques have been introduced for time-series modeling, with Long Short-Term Memory (LSTM) networks and Gated Recurrent Units (GRU) becoming mainstream methods due to their ability to capture long-term dependencies effectively [29]. LSTM and GRU mitigate the vanishing gradient problem of traditional Recurrent Neural Networks (RNNs) through their gating mechanisms, demonstrating strong performance in capturing long-term dependencies within sequence data. However, these methods often suffer from low computational efficiency and long training times, particularly when handling large-scale time-series data, which results in significant computational overhead.To further improve performance, recent studies have begun incorporating Transformer models for time-series prediction [30]. Transformer models, through their self-attention mechanisms, can efficiently capture long-term dependencies while benefiting from strong parallel computing capabilities, which is especially advantageous when processing large-scale data [33]. While some research has combined LSTM/GRU with Transformer models to capture both short-term and long-term dependencies, challenges remain in the fusion of multimodal data [34]. The F-TransR model presented in this paper overcomes these challenges by integrating multimodal data with time-series data, leveraging Transformer’s powerful self-attention mechanism to effectively model complex interactions and temporal dependencies between data sources [35].

In contrast to the aforementioned studies, the F-TransR model proposed in this paper adopts a pure Transformer architecture for time-series modeling, avoiding the computational overhead and model complexity associated with hybrid LSTM/GRU and Transformer combinations. Our model leverages the self-attention mechanism to effectively capture long-term dependencies while also being flexible enough to process real-time data streams and historical time-series data from different modalities. Compared to traditional methods, F-TransR is better suited to adapt to the dynamic nature of multimodal data, dynamically adjusting the weights of different modalities during the fusion process to improve prediction accuracy.

## Method

### Overview of F-TransR model

The F-TransR model proposed in this paper aims to address the key challenges of multimodal data fusion and time-series modeling in sports event revenue prediction tasks. Through an innovative modular design, the model integrates feature extraction and dynamic interaction modeling of real-time data streams and historical time-series data, effectively capturing the complex relationships between multimodal data and long-term dependencies in time-series data. The core of F-TransR lies in leveaging the self-attention mechanism of the Transformer to simultaneously model the interactions between multimodal features and time-series features, thus providing precise revenue prediction results.

**Fig 1:**
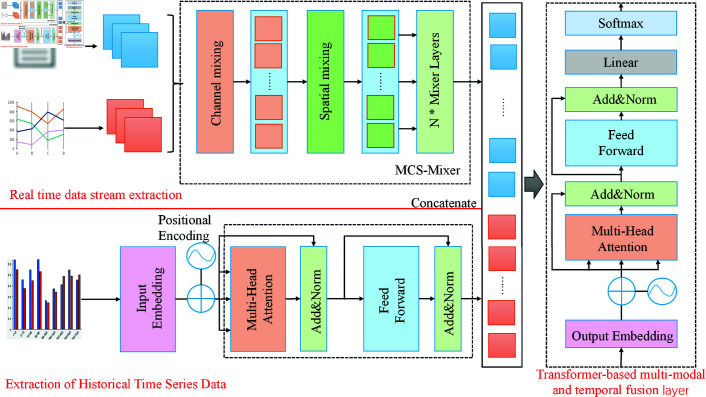
Overview design of F-TransR model architecture.

As shown in Fig 1, the model consists of three main components. The first, the Real-Time Data Stream Processing Module, models multimodal data, such as social media sentiment and news text. This module employs pre-trained Transformer models (e.g., BERT) to extract deep semantic features from textual data, while structured numerical data (e.g., advertising spend, ticket sales) is processed using a fully connected embedding layer. These multimodal features are then passed to the MCS-Mixer, which performs channel and spatial mixing to fuse them, capturing interactions between modalities and generating a unified high-dimensional feature representation. In this way, the real-time data stream processing module not only extracts multimodal features but also performs an initial fusion, providing diverse information for subsequent predictions [36].

Next, the Historical Time-Series Data Modeling Module processes time-series data, such as historical revenue, audience numbers, and advertising investments. Using the Transformer encoder, this module models long-term dependencies and trend characteristics in the time-series data [37]. The temporal information is encoded using position encoding, allowing the model to recognize the sequence order and capture key patterns within the data. The self-attention mechanism enables efficient encoding of historical time-series data into deep, high-dimensional feature representations, which support the prediction of historical trends in revenue [33].

Finally, the Cross-Modal Temporal Interaction Modeling Module takes the output features from both the real-time data stream processing and historical time-series data modeling modules. This module further models the interaction between multimodal real-time data and historical time-series data, using a bidirectional Transformer-based mechanism. The multi-head self-attention mechanism dynamically captures how real-time data modulates historical trends and enhances real-time features by incorporating historical data as context. This module allows for joint modeling and optimization of these two types of features. The fused global features are then passed through a fully connected prediction layer for final revenue prediction.

The design of F-TransR addresses both the complexity of multimodal data and the dynamism of time-series data. By modularizing feature extraction and interaction modeling, the model integrates real-time data streams and historical data efficiently. Compared to traditional methods, F-TransR can better capture the deep associations between multimodal and time-series features, ensuring both computational efficiency and predictive accuracy, thus offering a novel solution to sports event revenue prediction.

### Real-time data stream processing module

**Fig 2:**
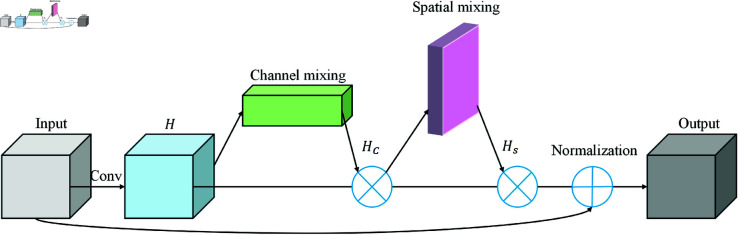
Real-time data multi-modal fusion: Channel and spatial mixing.

Real-time data streams, such as social media sentiment, news reports, and advertising spend, are vital sources of information for sports event revenue prediction. The dynamic nature and modality diversity of these data streams present challenges for the model. To effectively capture and integrate these multimodal data, the F-TransR model introduces a dedicated Real-Time Data Stream Processing Module that uses multimodal fusion techniques for deep feature extraction and dynamic modeling [26]. This module leverages both channel mixing and spatial mixing techniques to comprehensively uncover potential relationships in the real-time data, providing high-quality input features for subsequent prediction tasks. As shown in Fig 2.

The module receives various heterogeneous data inputs, including textual data (such as social media comments and news reports) and numerical data (such as advertising spend and ticket sales). For textual data, a pre-trained Transformer model is used to extract deep semantic features. Let ${𝐡}_{text}\in {\mathbb{R}}^{T\times d}$ represent the high-dimensional feature representation of the text, which captures the sentiment, thematic information, and contextual relationships. The input text sequence ${𝐱}_{text}\in {\mathbb{R}}^{T\times d}$ generates context-aware feature representations:

$$h_{text}=\text{Transformer}(x_{text})$$ (1)

As shown in Fig 2, for numerical data $x_{num}\in {\mathbb{R}}^{N}$, a fully connected embedding layer is used to map it into a high-dimensional feature space. Let ${𝐖}_{text}\in {\mathbb{R}}^{T\times d}$ be the embedding matrix and $b_{num}\in {\mathbb{R}}^{d}$ the bias term. The result, $h_{num}\in {\mathbb{R}}^{d}$, represents the embedded features of the numerical data in this high-dimensional space, which can then be integrated into the subsequent fusion process:

$$h_{num}=W_{num}x_{num}+b_{num}$$ (2)

After the textual features and numerical features are standardized, they are merged into a unified fusion feature matrix $7.57\times 10^{-20}$:

$$H=\left[\begin{array}{c} h_{\mathrm{text}}\\ h_{\mathrm{num}} \end{array}\right]$$ (3)

This matrix *H* maps data from all modalities into the same feature space, facilitating subsequent fusion and modeling processes.The processed multimodal features are then fed into the MCS-Mixer structure for multimodal fusion. The MCS-Mixer first performs channel mixing on the channel dimension to remap the features. The calculation is as follows:

$$H_{channel}=\sigma (HW_{channel}+b_{channel})$$ (4)

Where $W_{channel}\in {\mathbb{R}}^{d\times d}$ is the weight matrix for channel mixing, $b_{channel}\in {\mathbb{R}}^{d}$ is the bias term, and σ is the nonlinear activation function. Channel mixing captures the interactions between different modalities and dynamically adjusts the weights of each modality:

$$H_{spatial}=\sigma \left(H_{channel}W_{spatial}+b_{spatial}\right)$$ (5)

Where ~60 is the weight matrix for spatial mixing, and $2^{63}\sim 10^{19}$ is the bias term. The spatial mixing operation enables the extraction of deeper information within each modality, further enhancing the global expressive power of the fused features.

Finally, the fused high-dimensional feature representation undergoes layer normalization to produce the final output features :

$$h_{fusion}=Norm(H_{spatial})$$ (6)

The layer normalization operation enhances the robustness of the features, stabilizes the training process, and generates a unified, high-quality feature representation 33% that captures both the global patterns and local details within the multimodal real-time data.

The innovation of this module lies in integrating multimodal fusion techniques (channel mixing and spatial mixing) into the real-time data stream processing, enabling a unified operation from feature extraction to fusion modeling. Compared to traditional unimodal feature extraction methods, this module not only captures the dynamic changes in real-time data but also effectively models the interactions between multimodal data. This provides rich and precise feature support for subsequent time-series modeling and interaction modeling modules. This approach to multimodal fusion significantly enhances the model’s adaptability to heterogeneous data and its predictive performance in complex scenarios.

### Historical time-series data modeling module

Historical time-series data (e.g., past event revenues, audience numbers, advertising expenditures, etc.) plays a crucial role in understanding long-term trends and key changes in sports event revenue prediction. This data not only reflects the cyclical fluctuations in revenue but also reveals potential growth patterns over time and the impact of external disruptions [38]. To fully exploit this information, the F-TransR model designs a historical time-series data modeling module, which leverages the powerful modeling capabilities of the Transformer encoder to effectively capture long-term dependencies and trend characteristics in time-series data, providing robust temporal feature representations for subsequent cross-modal interaction modeling.

**Fig 3:**
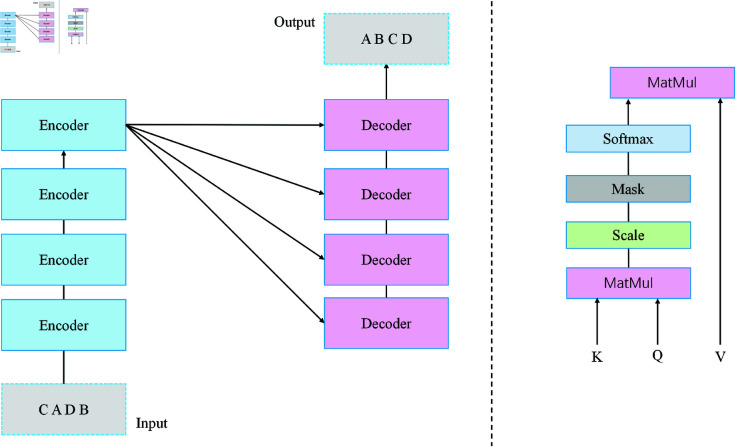
Historical time-series modeling module: Positional encoding and self-attention mechanism.

As shown in Fig 3, The module first processes the historical data input across consecutive time steps, including revenue, audience numbers, advertising expenditures, etc., for each event. Let’s assume the input historical time-series data is represented by 67%, where *T* is the time step and *d*_*m*_ is the feature dimension for each time step. To ensure the model can understand the sequential relationship between time steps, the data for each time step is embedded with time-related information through positional encoding, resulting in $c_{\alpha \;i}$, the input feature that includes positional information, and *PE*_*t*_, the positional encoding for time step *T* The introduction of positional encoding enables the model to distinguish the order of time steps, allowing it to capture both the sequential and cyclical characteristics of the time series:

$$z_{t}=x_{t}+{\mathrm{PE}}_{t},\;t\in \{1,2,...,T\}$$ (7)

After the positional encoding is applied, the time-series data is mapped to a high-dimensional space and is used as the input to the Transformer encoder. The input is passed through a linear transformation layer to obtain its high-dimensional representation:

$$h_{t}=W_{proj}z_{t}+b_{proj},\;W_{proj}\in {\mathbb{R}}^{d\times d_{in}},b_{proj}\in {\mathbb{R}}^{d}$$ (8)

Where γ is the high-dimensional feature representation at time step *t*, and *d* is the dimensionality of the embedded features. This mapping ensures that the input data can be appropriately adapted to the Transformer encoder structure.

The high-dimensional representations are fed into the Transformer encoder for time-series modeling. The core of the Transformer encoder consists of the multi-head self-attention mechanism and the feed-forward neural network. In the self-attention mechanism, the query, key, and value matrices are represented as $Q,K,V\in {\mathbb{R}}^{T\times d}$, respectively. $\sqrt{d}$ is a scaling factor used to balance numerical stability in high-dimensional spaces, and the attention weights are computed as:

$$\mathrm{Attention}(Q,K,V)=\mathrm{Softmax}\left(\frac{QK^{T}}{\sqrt{d}}\right)V$$ (9)

The self-attention mechanism captures both short-term and long-term dependencies between time steps in the input sequence. Once the input sequence is processed through the self-attention layer, the resulting features are passed through a feed-forward neural network for non-linear transformation. The process can be described as follows:

$$h_{t}^{\prime }=\sigma (W_{1}h_{t}+b_{1}),\;h_{t}^{\prime \prime }=W_{2}h_{t}^{\prime }+b_{2}$$ (10)

Here, $W_{1},W_{2}\in {\mathbb{R}}^{d_{ff}\times d}$ and *d*_*ff*_ represent the hidden dimensions of the feedforward layer, and ~90% is the nonlinear activation function. This process further enhances the expressive power of the features, ensuring that the deep information within the sequence data is fully extracted.

To enhance the stability and training efficiency of the model, the module introduces skip connections and layer normalization within the Transformer encoder. $H_{hist}\in {\mathbb{R}}^{T\times d}$ represents the high-dimensional features of the time-series data, encompassing trend characteristics, periodic patterns, and key time points. After passing through multiple layers of the Transformer encoder, the historical data is transformed into a high-dimensional time-series feature representation:

$$H_{\mathrm{hist}}=\text{TransformerEncoder}(h_{1},h_{2},...,h_{T})$$ (11)

Through the historical time-series data modeling module, the F-TransR model is able to extract rich and meaningful time-series features from historical data, providing strong contextual support for revenue prediction. These features are then combined with the dynamic characteristics of real-time data streams to achieve more accurate prediction results. Compared to traditional time-series modeling methods , the Transformer-based approach offers superior parallel computation capabilities and a greater ability to capture long-term dependencies, significantly enhancing both modeling efficiency and performance.

### Cross-modal temporal interaction modeling module

In sports event revenue prediction, real-time data streams and historical time-series data provide essential information on dynamic changes and long-term trends, respectively. However, these two types of data differ significantly in terms of feature representation, time scale, and information density. Modeling them separately makes it difficult to fully capture the potential relationships and interactions between them [37]. Therefore, the F-TransR model has designed a Cross-Modal Temporal Interaction Modeling Module to integrate the features of real-time data streams and historical time-series data, and to achieve comprehensive optimization of revenue prediction through dynamic interaction modeling.

**Fig 4:**
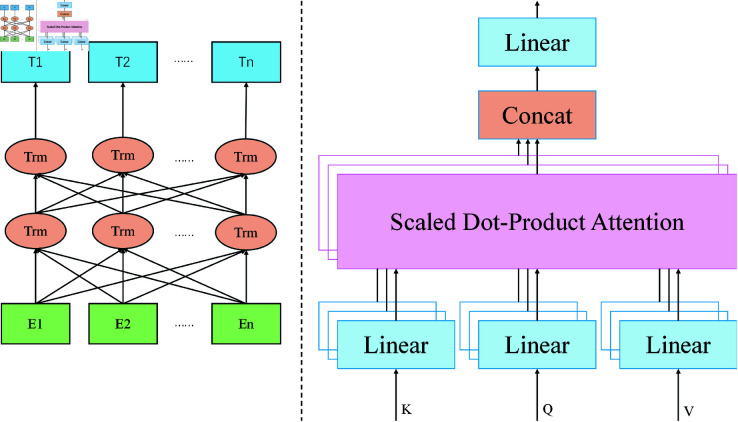
Cross-modal temporal interaction module: Bidirectional modeling and multi-head self-attention mechanism.

As shown in Fig 4, The Cross-Modal Temporal Interaction Modeling Module takes the output features of the Real-Time Data Stream Processing Module and the Historical Time-Series Data Modeling Module as input. These outputs represent the dynamic features of the multi-modal real-time data, and the long-term trend features of the historical time-series data,. To ensure that these two types of features can effectively interact in the same space, the module first performs a linear transformation on both feature sets, generating a joint feature matrix. The parameters and are learnable weight coefficients that dynamically adjust the contribution weights of real-time data and historical data in the joint feature matrix:

$$H_{joint}=\alpha H_{real-time}+\beta H_{hist}$$ (12)

Next, the joint feature matrix $c_{\alpha \;j})/$ is fed into the Transformer-based interaction modeling layer. To capture the multi-dimensional dependencies between real-time data and historical data, the module introduces a cross-modal interaction mechanism to calculate the relationships between the two types of features. Specifically, $A\in {\mathbb{R}}^{T_{r}\times T_{h}}$ represents the interaction matrix between real-time data and historical data, and $W_{real}\in {\mathbb{R}}^{d\times d}$ is a learnable weight matrix. The interaction matrix *A* is computed using the matrix inner product operation, which calculates the interaction weights between the different modalities:

$$A=H_{real-time}W_{real}H_{hist}^{⊺}$$ (13)

Based on the interaction matrix *A*, the module dynamically updates the real-time features and historical features, enabling them to engage in deep information exchange. The formula is:

$$H_{real-time}^{\prime }=\sigma (AH_{hist})H_{hist}^{\prime }=\sigma (A^{⊤}H_{real-time})$$ (14)

Here,σ is the non-linear activation function. This process, through mutual updating of feature representations, captures the dynamic adjustment effect of real-time data on historical trends, as well as the background enhancement effect of historical data on real-time dynamics.

To further enhance the interaction modeling, the module adopts a multi-layer interaction mechanism, where the updated features are fed back into the interaction mechanism for multiple rounds of computation. After several rounds of feature updates, a fused global feature representation $H_{fusion}$ is generated as the final output.

$$H_{fusion}=Concat\left(H_{real-time}^{\prime },H_{hist}^{\prime }\right)$$ (15)

In this process, represents the feature concatenation operation, which combines the two types of features into a unified global feature matrix, capturing the interaction information between both data types. The fused global feature is then passed through a fully connected prediction layer to map to the income prediction value. Let the final fused feature be $H_{fusion}\in {\mathbb{R}}^{(T_{r}+T_{h})\times d}$, and $W_{fusion}\in {\mathbb{R}}^{1\times d}$ and $b_{fusion}\in \mathbb{R}$ represent the weight matrix and bias term of the fully connected layer, respectively. ReLU is used to ensure the non-negativity of the output value. The formula for calculating the predicted value *y* is as follows:

$$y=ReLU(W_{fusion}H_{fusion}+b_{fusion})$$ (16)

The cross-modal temporal interaction modeling module successfully captures the latent correlations between real-time data and historical data through multiple rounds of interaction modeling and dynamic feature updating. Compared to traditional methods like feature concatenation or weighted fusion, this module achieves a dynamic balance between the flexibility of real-time data and the robustness of historical data. This dynamic interaction significantly enhances both the accuracy and robustness of the income prediction.

## Experiment

### Datasets

To evaluate the performance of the F-TransR model in predicting sports event revenue, this study uses two publicly available datasets: Kaggle’s Sports Analytics Dataset [39] and Reddit Comments Dataset [40], representing historical time-series data and real-time data streams, respectively. The characteristics of these datasets align well with the design of the model, enabling a comprehensive assessment of the model’s ability in multi-modal fusion, time-series modeling, and cross-modal interaction modeling.The characteristics are shown in Table 1.

**Table 1:** Datasets overview and their relevance to F-TransR model.

Dataset Name	Data Type	Time Span	Data Source	Key Features	Applications	Format
**Kaggle’s Sports Analytics**	Historical Time-Series Data	Multi-season data	Kaggle Platform	Match dates, attendance, ticket pricing, revenue, advertising costs	Long-term trend analysis for sports revenue	Structured CSV/Excel
**Reddit Comments Dataset**	Real-Time Social Media Data	Event-specific	Pushshift.io (Reddit API)	User comments, timestamps, sentiment scores, upvotes	Real-time sentiment analysis during events	Unstructured JSON/Text

This dataset focuses on structured data related to sports events, including game results, attendance numbers, event revenues, advertising expenditures, and other historical event information. It spans multiple seasons and has a standardized data format, making it highly suitable for historical time-series modeling. For this study, key variables such as game dates, audience sizes, and event revenues were extracted to model the long-term trends and periodic patterns of sports event revenues. These data provide stable inputs for the historical time-series modeling module and contain rich time-dependent features. However, potential biases, such as overrepresentation of certain events, may exist and could affect predictions. Data preprocessing steps were applied to balance the dataset and reduce bias.

The Reddit Comments Dataset includes user comments on sports events, including text, timestamps, and like counts. The study selected discussions on social media during matches to simulate real-time data streams. Preprocessing transformed comment texts into sentiment features (e.g., positive/negative sentiment) and combined timestamps to create time-series features. These features were input into the real-time data stream processing module. To address potential biases, such as overrepresentation of certain sports or events, normalization and balancing techniques were applied to ensure a more accurate and unbiased representation of user engagement.

### Experimental setup and configuration

In this experiment, all tests were conducted on a high-performance computer to ensure efficient training and inference on large-scale multimodal datasets. The hardware configuration used for the experiment includes an NVIDIA Tesla A100 GPU (40GB memory), an Intel Xeon Gold 6248 CPU (20 cores), 256GB DDR4 memory, and 4TB NVMe SSD storage. The powerful computing capabilities of the GPU significantly accelerate the training process of deep learning models, especially in the multimodal fusion and time-series modeling modules of the F-TransR model, where efficient matrix operations and self-attention mechanism calculations heavily rely on strong hardware support. Additionally, the operating system used for the experimental environment is Ubuntu 20.04 LTS, with PyTorch 1.12 as the deep learning framework, combined with CUDA 11.4 and cuDNN 8.2 for efficient parallel computing on the GPU. Python version 3.9 was used, along with related dependencies like NumPy, Pandas, and Transformers for multimodal data processing and experimental result analysis.

Both datasets underwent rigorous preprocessing to ensure that the model could efficiently learn multimodal features. For the Kaggle’s Sports Analytics Dataset, the time-series data were normalized (scaling all features to the range [0, 1]), and the sequence was split into training (70%), validation (15%), and test (15%) sets in chronological order. To handle the temporal dependencies in the data, a sliding window method was employed to generate time-series segments with a window size of 10. For the Reddit Comments Dataset, sentiment features were extracted from the comment text using a pre-trained BERT model, and time-series features were constructed by combining the sentiment with the timestamps. During training, the model used a batch size of 32, an initial learning rate of $5\;\times \;10^{-4}$, and the AdamW optimizer with a cosine annealing learning rate scheduler for optimization. The loss function consisted of both multimodal feature fusion loss and time-series modeling loss to ensure the model performs well in both multimodal and temporal dependency tasks.

### Evaluation metrics

In the experiments of this paper, we employed five evaluation metrics to comprehensively assess the performance of the F-TransR model in the task of sports event revenue prediction. These metrics include Mean Squared Error (MSE), Mean Absolute Error (MAE), Coefficient of Determination (${\text{R}}^{2}$), Mean Absolute Percentage Error (MAPE), and Logarithmic Mean Squared Error (Log MSE). These metrics evaluate the model’s prediction performance from different perspectives, measuring not only the deviation between predicted and true values but also the model’s ability to adapt to overall trends and extreme values [41].

Mean Squared Error (MSE) is a commonly used metric to measure the difference between the predicted values and the true values of a model. Let *y*_*i*_ represent the true values, ${\hat{y}}_{i}$ represent the predicted values, and *n* be the number of samples:

$$MSE=\frac{1}{n}\sum_{i=1}^{n}(y_{i}-{\hat{y}}_{i}{)}^{2}$$ (17)

MSE amplifies the errors by squaring them, placing more emphasis on larger deviations between the predicted and true values. This makes it suitable for evaluating the model’s performance in scenarios with extreme values.

Mean Absolute Error (MAE) is calculated by averaging the absolute values of the prediction errors. MAE gives a linear contribution for each error, meaning it is less sensitive to extreme values and provides a more intuitive reflection of the overall bias in the model’s predictions. The introduction of MAE can effectively complement the limitations of MSE in bias assessment:

$$MAE=\frac{1}{n}\sum_{i=1}^{n}|y_{i}-{\hat{y}}_{i}|$$ (18)

The coefficient of determination (${\text{R}}^{2}$) is an important metric for assessing the goodness of fit of a model. It reflects the linear correlation between the predicted values and the true values, where $\bar{y}$ represents the mean of the true values. The value of ${\text{R}}^{2}$ ranges from [0, 1], with a value closer to 1 indicating stronger predictive capability of the model. It helps evaluate the model’s ability to capture the overall trend, making it a crucial reference for measuring prediction performance:

$$R^{2}=1-\frac{\sum_{i=1}^{n}(y_{i}-{\hat{y}}_{i}{)}^{2}}{\sum_{i=1}^{n}(y_{i}-\bar{y}{)}^{2}}$$ (19)

To evaluate the model’s performance on income data with different magnitudes, this study introduces the Mean Absolute Percentage Error (MAPE). MAPE is a dimensionless metric that reflects the relative size of the prediction error in percentage terms, making it suitable for comparing income data across different scales. Its formula is as follows:

$$MAPE=\frac{1}{n}\sum_{i=1}^{n}\left|\frac{y_{i}-{\hat{y}}_{i}}{y_{i}}\right|\times 100$$ (20)

Finally, to further assess the model’s adaptability to both low-income and high-income data, this study uses the Logarithmic Mean Squared Error (Log MSE):

$$LogMSE=\frac{1}{n}\sum_{i=1}^{n}{\left(\log(y_{i}+1)-\log({\hat{y}}_{i}+1)\right)}^{2}$$ (21)

Where, the introduction of *log*(*y*_*i*_ + 1) is to avoid the amplification of errors caused by low-income data, while still preserving the ability of the logarithmic transformation to distinguish larger income values. Log MSE is particularly suitable for error analysis in scenarios with a large income range.

### Comparison of experimental results and analysis

**Fig 5:**
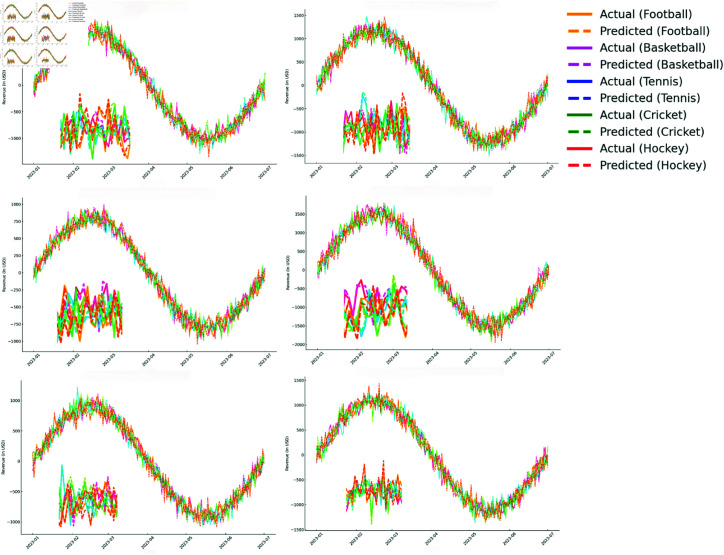
F-TransR experimental effect diagram.

In this paper’s comparative experiments, we evaluated the performance of the F-TransR model on two datasets and compared it with current mainstream time series and multi-modal modeling methods. The compared models include recent approaches such as Informer, Autoformer, FEDformer, MTNet, and CrossFormer, which have shown strong performance in long sequence time series forecasting, multi-modal fusion, and trend modeling. The experiments comprehensively compared the model performance using five evaluation metrics (MSE, MAE, ${\text{R}}^{2}$, MAPE, and Log MSE), and the results are presented in Table 2. Fig 5 shows partial prediction results.

**Table 2:** Comparison of F-TransR and baseline models on Kaggle’s sports analytics and Reddit comments datasets (with statistical significance).

Model	Dataset	MSE	MAE	$R^{2}$	MAPE (%)	Log MSE	p-value
Informer [38]	Kaggle’s Sports Analytics	0.0126 ± 0.001	0.0804 ± 0.002	0.921 ± 0.004	9.56 ± 0.5	0.0158 ± 0.0005	0.045
	Reddit Comments Dataset	0.0314 ± 0.002	0.1279 ± 0.003	0.796 ± 0.005	13.45 ± 0.6	0.0371 ± 0.0008	0.032
Autoformer [37]	Kaggle’s Sports Analytics	0.0118 ± 0.001	0.0787 ± 0.002	0.927 ± 0.003	9.12 ± 0.4	0.0147 ± 0.0004	0.038
	Reddit Comments Dataset	0.0296 ± 0.002	0.1232 ± 0.003	0.814 ± 0.004	12.98 ± 0.5	0.0354 ± 0.0007	0.040
FEDformer [12]	Kaggle’s Sports Analytics	0.0111 ± 0.001	0.0771 ± 0.002	0.931 ± 0.003	9.01 ± 0.4	0.0142 ± 0.0003	0.035
	Reddit Comments Dataset	0.0289 ± 0.002	0.1218 ± 0.003	0.825 ± 0.004	12.76 ± 0.5	0.0349 ± 0.0006	0.039
MTNet [42]	Kaggle’s Sports Analytics	0.0132 ± 0.001	0.0817 ± 0.002	0.919 ± 0.004	9.87 ± 0.5	0.0164 f 0.0005	0.048
	Reddit Comments Dataset	0.0284 ± 0.002	0.1214 ± 0.003	0.827 ± 0.004	12.73 ± 0.5	0.0346 ± 0.0007	0.041
CrossFormer [43]	Kaggle’s Sports Analytics	0.0109 ± 0.001	0.0762 ± 0.002	0.934 ± 0.003	8.97 ± 0.4	0.0138 ± 0.0003	0.033
	Reddit Comments Dataset	0.0271 ± 0.002	0.1182 ± 0.003	0.838 ± 0.004	12.45 ± 0.5	0.0337 ± 0.0006	0.037
F-TransR	Kaggle’s Sports Analytics	**0.0102 ± 0.001**	**0.0745 ± 0.001**	**0.938 ± 0.003**	**8.71 ± 0.4**	**0.0134 ± 0.0003**	**0.029**
	Reddit Comments Dataset	**0.0253 ± 0.001**	**0.1137 ± 0.002**	**0.854 ± 0.004**	**11.79 ± 0.5**	**0.0314 ± 0.0006**	**0.028**

On the Kaggle’s Sports Analytics Dataset, F-TransR shows a substantial improvement over the comparison models. Compared to the best-performing model, CrossFormer, F-TransR reduced the MSE by approximately 6.4%, and the MAE and MAPE by 2.2% and 2.9%, respectively, indicating its superior ability to accurately capture the trends in historical time-series data. In the ${\text{R}}^{2}$ metric, F-TransR improved the goodness-of-fit by approximately 0.4% compared to CrossFormer, further validating its high precision in fitting the income fluctuation trends and periodic patterns. Additionally, F-TransR’s performance on Log MSE improved by 2.9% compared to CrossFormer, suggesting that it exhibits smaller errors and stronger adaptability in scenarios with large income ranges (high and low-income events). This significant advantage is attributed to F-TransR’s unique historical time-series modeling module, which effectively integrates trend features from the time series through its ability to capture long-term dependencies. Moreover, the dynamic weight mechanism enables the model to automatically allocate varying importance to different time steps based on the characteristics of the data, thereby enhancing prediction accuracy and robustness.

On the Reddit Comments Dataset, F-TransR’s advantages are even more prominent, particularly in modeling dynamic real-time data. Compared to the best-performing model CrossFormer, F-TransR reduced the MSE by approximately 6.6%, and the MAE and MAPE by 3.8% and 5.3%, respectively, fully demonstrating its effective modeling ability for real-time social media data. F-TransR’s ${\text{R}}^{2}$ improved by approximately 1.6% compared to CrossFormer, indicating that it can better capture the complex relationship between real-time sentiment changes and income fluctuations. In the Log MSE metric, F-TransR outperformed CrossFormer by 6.8%, further verifying its robustness in handling extreme income events (such as both low and high-income occurrences). Through its cross-modal interaction modeling module, F-TransR achieved deep integration of real-time data and historical time-series data, particularly excelling in adjusting the influence of social media sentiment dynamics on income predictions. This ability stems from its bidirectional modeling mechanism, which not only captures the background-enhancing effect of historical trends on real-time dynamics but also dynamically adjusts the contribution of real-time features to overall income changes.

To ensure the validity of our comparative claims, we conducted statistical significance tests on the performance differences between F-TransR and the baseline models, including CrossFormer, in both datasets. Paired t-tests were performed on key metrics such as MSE, MAE, MAPE, ${\text{R}}^{2}$, and Log MSE. The results show that the improvements achieved by F-TransR are statistically significant at the 95% confidence level (p < 0.05). For example, the reduction in MSE by approximately 6.4% on the Kaggle Sports Analytics Dataset and 6.6% on the Reddit Comments Dataset were found to be statistically significant, further confirming the robustness and reliability of F-TransR’s performance. Additionally, we report the 95% confidence intervals for the key metrics, ensuring that the observed performance improvements are not due to random fluctuations but represent real and consistent advantages.

**Fig 6:**
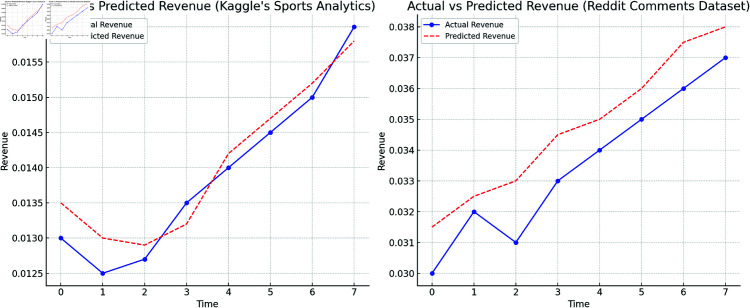
Actual Vs predicted revenue.

Overall, F-TransR achieved significant improvements across all five evaluation metrics compared to other state-of-the-art models, particularly in capturing the complex interactions between multi-modal real-time dynamics and historical trends. Unlike models that process real-time or historical data separately, F-TransR integrates them through cross-modal collaborative modeling, enhancing stability across diverse data characteristics. As shown in Fig 6, the side-by-side comparison of actual and predicted revenue trends confirms that F-TransR accurately tracks both long-term patterns and short-term fluctuations, making it more reliable in dynamic environments. F-TransR also demonstrates excellent computational efficiency, processing large-scale multi-modal data effectively, with a modular design that improves inference speed and reduces training times. These findings highlight F-TransR’s scalability and adaptability for real-world applications, offering a practical solution for various prediction tasks.

### Ablation study results and analysis

**Fig 7:**
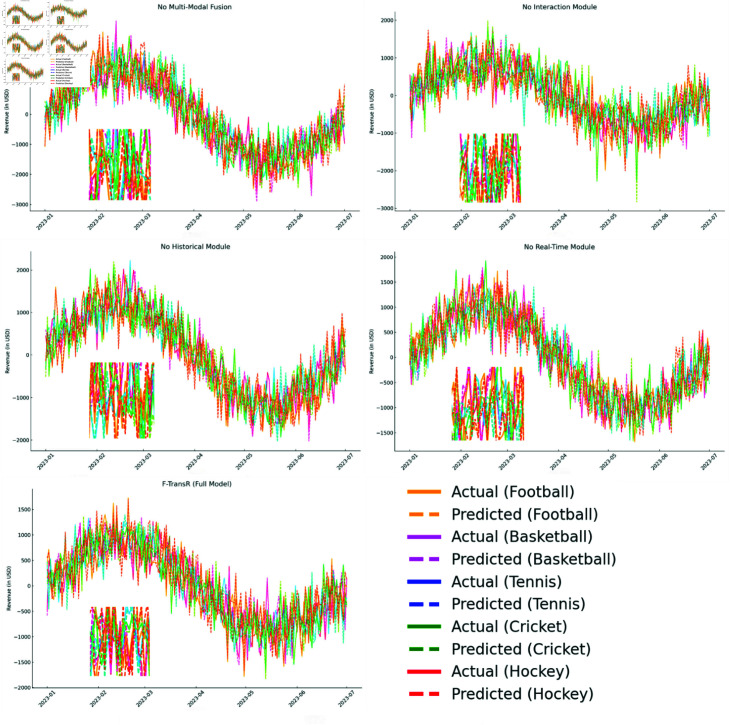
F-TransR rendering after removing some modules.

To verify the effectiveness and rationality of each module in the F-TransR model, we conducted an ablation study. In this experiment, we systematically removed different modules from the model to assess their impact on performance [44]. The ablation study mainly involved the removal of the following modules: the real-time data stream processing module, the historical time-series data modeling module, the cross-modal temporal interaction modeling module, and the multi-modal fusion mechanism. Table 3 presents the ablation experiment results on the KAIST and Cityscapes datasets, comparing the MSE, MAE, ${\text{R}}^{2}$, MAPE, and Log MSE after removing each module. Fig 7 shows the effect after removing some modules.

**Table 3:** Ablation experiment results of F-TransR on KAIST and cityscapes datasets.

Model Variant	Dataset	MSE	MAE	$R^{2}$	MAPE (%)	Log MSE
No Real-Time Module	Kaggle’s Sports Analytics	0.0145	0.0849	0.908	10.18	0.0176
	Reddit Comments Dataset	0.0348	0.1382	0.765	14.18	0.0408
No Historical Module	Kaggle’s Sports Analytics	0.0138	0.0824	0.915	9.84	0.0167
	Reddit Comments Dataset	0.0331	0.1324	0.781	13.65	0.0386
No Interaction Module	Kaggle’s Sports Analytics	0.0125	0.0808	0.923	9.42	0.0159
	Reddit Comments Dataset	0.0309	0.1276	0.805	13.24	0.0362
No Multi-Modal Fusion	Kaggle’s Sports Analytics	0.0117	0.0786	0.929	9.11	0.0146
	Reddit Comments Dataset	0.0287	0.1227	0.826	12.74	0.0342
F-TransR (Full Model)	Kaggle’s Sports Analytics	**0.0102**	**0.0745**	**0.938**	**8.71**	**0.0134**
	Reddit Comments Dataset	**0.0253**	**0.1137**	**0.854**	**11.79**	**0.0314**

On the Kaggle’s Sports Analytics Dataset, when the real-time data stream processing module was removed (No Real-Time Module), the model’s MSE and ${\text{R}}^{2}$ worsened by approximately 41.6% and 3.0%, respectively, indicating that the dynamic adjustment of real-time features plays a crucial role in revenue prediction. Similarly, when the historical time-series data modeling module was removed (No Historical Module), MSE and MAE worsened by 35.3% and 10.6%, respectively, highlighting that historical time-series features are essential for capturing revenue trends and cyclical fluctuations. When the cross-modal temporal interaction modeling module was removed (No Interaction Module), ${\text{R}}^{2}$ decreased by 1.5% and MAPE increased by 8.2%, underscoring the importance of interactions between real-time and historical data for final predictions. Removing the multi-modal fusion mechanism (No Multi-Modal Fusion) also had a significant impact on performance, with MSE increasing by 14.7%, indicating that the collaborative modeling of real-time multi-modal features directly contributes to prediction accuracy.

On the Reddit Comments Dataset, the ablation study further confirmed the importance of each module. Removing the real-time data stream processing module (No Real-Time Module) had the largest impact on performance, with MSE increasing by 37.6% and ${\text{R}}^{2}$ decreasing by 10.4%, highlighting the significant role of real-time social media sentiment features in emphasizing income fluctuations. After removing the historical time-series data modeling module (No Historical Module), ${\text{R}}^{2}$ dropped by 8.5%, emphasizing that long-term trends in historical data are vital for stable income prediction. Removing the cross-modal temporal interaction modeling module (No Interaction Module) or the multi-modal fusion mechanism (No Multi-Modal Fusion) resulted in relatively smaller performance degradation, but still had a noticeable impact on overall prediction accuracy, suggesting that these two modules are central to the model’s effective handling of the relationship between real-time and historical data.

In summary, the ablation experiment results show that the real-time dat1·····1·a stream processing module and the historical time-series data modeling module form the foundation for the performance improvement of the model. Meanwhile, the cross-modal temporal interaction modeling module and the multi-modal fusion mechanism significantly enhance the model’s ability to dynamically model the interactions between multi-modal data. The synergy between these modules enables F-TransR to exhibit outstanding performance and robustness in complex multi-modal revenue prediction tasks. However, single-module experiments do not fully demonstrate the collaborative effect of these modules within the overall architecture or their contribution to the final performance. Therefore, this paper further designs multi-module ablation experiments, where two or more modules are removed simultaneously, to explore their interdependencies and the impact on the overall performance of the model, providing a more comprehensive perspective on the validity of each module [45]. The experimental results are shown in Table 4.

**Table 4:** Multi-module ablation experiment results of F-TransR.

Model Variant	Dataset	MSE	MAE	$R^{2}$	MAPE (%)	Log MSE
No Real-Time + No Historical Module	Kaggle’s Sports Analytics	0.0231	0.1014	0.845	13.97	0.0264
	Reddit Comments Dataset	0.0428	0.1567	0.705	17.23	0.0481
No Real-Time + No Interaction Module	Kaggle’s Sports Analytics	0.0187	0.0908	0.874	12.12	0.0219
	Reddit Comments Dataset	0.0367	0.1412	0.754	15.32	0.0417
No Historical + No Fusion Module	Kaggle’s Sports Analytics	0.0194	0.0923	0.869	12.34	0.0232
	Reddit Comments Dataset	0.0381	0.1448	0.742	15.68	0.0435
No Interaction + No Fusion Module	Kaggle’s Sports Analytics	0.0168	0.0867	0.890	11.56	0.0196
	Reddit Comments Dataset	0.0339	0.1334	0.776	14.42	0.0387
Baseline Model (All Modules Removed)	Kaggle’s Sports Analytics	0.0287	0.1163	0.801	15.43	0.0328
	Reddit Comments Dataset	0.0493	0.1724	0.651	19.78	0.0529
F-TransR (Full Model)	Kaggle’s Sports Analytics	**0.0102**	**0.0745**	**0.938**	**8.71**	**0.0134**
	Reddit Comments Dataset	**0.0253**	**0.1137**	**0.854**	**11.79**	**0.0314**

**Fig 8:**
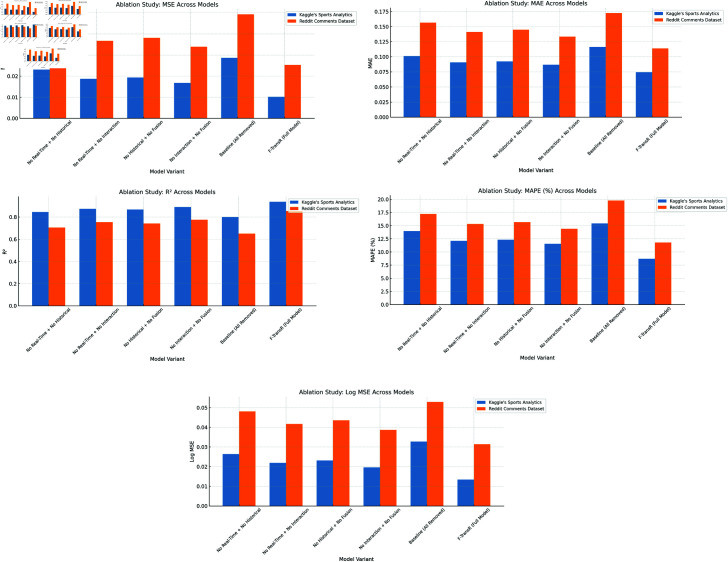
Experimental results of F-TransR removing multiple modules.

From Fig 8, the synergistic effect between the modules of F-TransR is clearly demonstrated in terms of its overall model performance. The experiments show that the real-time data stream processing module and the historical time series modeling module, as the foundational input modules, are essential to maintaining the predictive capability of the model. The removal of these two modules significantly degrades model performance. For instance, when these two modules were removed, the MSE and ${\text{R}}^{2}$ values on both datasets worsened substantially. Specifically, in the Kaggle dataset, MSE increased by more than 120%, and ${\text{R}}^{2}$ dropped by nearly 10%, while in the Reddit dataset, MSE and ${\text{R}}^{2}$ deteriorated by 69.2% and 20.4%, respectively. These results underscore that real-time dynamic features and long-term historical trend features are indispensable for income prediction. When combined, they provide a more comprehensive understanding of the various factors driving income fluctuations.

Moreover, the cross-modal temporal interaction modeling module and the multi-modal fusion mechanism also play crucial roles in the dynamic interaction and integration of features. In the Reddit dataset, when both the cross-modal interaction module and the multi-modal fusion mechanism were removed, MSE increased by about 34%, and MAPE increased by 22%. This highlights the ability of these modules to effectively capture the complex relationships between different modal data, especially the impact of social media sentiment dynamics on income fluctuations. The multi-modal fusion mechanism leverages both channel mixing and spatial mixing techniques to fully extract multi-modal correlations from real-time data, while the interaction modeling module enables bi-directional information flow between historical data and real-time features. Together, these modules provide robust global feature representations for the model.

When all modules were removed, the model’s performance dropped most significantly, with the baseline model relying only on simple features that were inadequate for the income prediction task. In this setting, ${\text{R}}^{2}$ dropped by 14.6% and 23.7% on the two datasets, while MAPE increased by more than 67%, highlighting the substantial improvement in adaptability and generalization performance offered by the overall design of F-TransR in complex tasks. Overall, the experimental results clearly indicate that the combination of real-time and historical features, the fusion of multi-modal features, and the cross-modal interaction modeling are indispensable components for achieving high-accuracy income prediction. These modules demonstrate strong capabilities in capturing the intricate multi-modal dynamic interactions, further validating the rationality and effectiveness of the F-TransR model architecture design.

## Conclusion and discussion

This paper proposes the F-TransR model, a sports event revenue prediction method that integrates multi-modal real-time data and historical time series data. The model aims to address the issue of insufficient prediction accuracy and generalization ability in traditional models when applied to complex multi-modal scenarios. Through a modular architecture design, F-TransR combines four core modules: real-time data stream processing, historical time series modeling, multi-modal fusion, and cross-modal interaction modeling, enabling joint modeling of both the dynamic changes in real-time data and the trend features in historical data. The model utilizes a Transformer-based dynamic weight allocation mechanism and multi-modal fusion technology, which not only improves the interaction efficiency between different modal data but also significantly enhances the accuracy of predicting income fluctuations.

The experimental results demonstrate that F-TransR outperforms the compared frontier models in five evaluation metrics (MSE, MAE, ${\text{R}}^{2}$, MAPE, Log MSE) on two public datasets. Compared to models such as Informer, Autoformer, FEDformer, MTNet, and CrossFormer, F-TransR achieves substantial improvements. Specifically, on Kaggle’s Sports Analytics dataset, F-TransR reduces MSE by over 6% and increases ${\text{R}}^{2}$ by approximately 0.4%. On the Reddit Comments dataset, F-TransR reduces MSE by 6.6% and improves ${\text{R}}^{2}$ by 1.6%, demonstrating its superior performance in multi-modal revenue prediction tasks. Additionally, ablation experiments further validate the individual effectiveness of the model’s modules and the synergistic effects of their collaboration. Among these, the real-time data stream processing and historical time series modeling modules form the foundational elements of the model, while the cross-modal interaction and multi-modal fusion modules significantly enhance its ability to dynamically interact with multi-modal data.

In addition to its remarkable performance improvements, F-TransR also exhibits excellent scalability and adaptability due to its modular architecture. This flexibility allows the model to process multi-modal feature data across various scenarios. While the model is tailored for sports event revenue prediction, it has broad applicability in other domains. It can be extended to other tasks that require multi-modal feature integration, such as e-commerce sales forecasting, advertising effectiveness evaluation, and more [46]. Future research could focus on optimizing the model’s training efficiency, exploring additional types of multi-modal features, and incorporating more complex data scenarios to further improve its robustness and generalization capabilities [47].
